# Vertebral heart scale measurements in Asiatic black bears (*Ursus thibetanus*) and sun bears (*Helarctos malayanus*)

**DOI:** 10.1371/journal.pone.0356816

**Published:** 2026-09-18

**Authors:** Nari Kim, Jacqui Esteban, Sorphea Phorn, Vi Le Tuong, Mengxiong Wangnengxiong, Zachary A. David, Matt Hunt, Dong-Hyuk Jeong

**Affiliations:** 1 College of Veterinary Medicine, Chungbuk National University, Sejong, Republic of Korea; 2 Free the Bears, Phnom Penh, Cambodia; 3 Free the Bears, Cat Tien, Vietnam; 4 Free the Bears, Luang Prabang, Laos; 5 Wildlife Center of Chungbuk, Cheongju, Republic of Korea; Wrocław University of Environmental and Life Sciences: Uniwersytet Przyrodniczy we Wroclawiu, POLAND

## Abstract

This retrospective study aimed to establish vertebral heart score (VHS) reference ranges for Asiatic black bear (*Ursus thibetanus*; ABB) and sun bear (*Helarctos malayanus*; SB), and to evaluate interspecific differences and the effects of biological variables on VHS measurement. Although cardiovascular disease is a common health concern for these species in captivity, objective radiographic reference ranges for cardiac size assessments are currently lacking. Thoracic radiographs from 46 clinically healthy sanctuary bears (22 ABBs, 24 SBs) were reviewed. VHS was measured on right lateral (RL) and dorsoventral (DV) views, and thoracic conformation was quantified using the thoracic depth-to-width (D/W) ratio. The influence of sex, age, and morphometric parameters (body weight, body condition score, and chest circumference) on VHS was evaluated within each species. In ABBs, the mean VHS was 10.65 ± 0.48 vertebrae (v) (95% range, 9.90 ‒ 11.67) on RL view and 10.82 ± 0.73 v (95% range, 8.90 ‒ 11.96) on DV views. Corresponding values in SBs were 10.68 ± 0.62 v (RL) (95% range, 9.19 ‒ 11.68) and 11.18 ± 0.67 v (DV) (95% range, 10.13 ‒ 12.28). No significant differences in VHS were observed between the two species, despite distinct differences in thoracic conformation; D/W ratio was 0.73‒1.00 (95% range) in ABBs and 0.59‒0.77 (95% range) in SBs. Sex, age, and morphometric variables were not significantly associated with VHS in either species. Despite significant interspecific variations in thoracic morphology, the VHS remained consistent between species and robust across various biological traits. These species-specific VHS reference ranges provide a practical radiographic tool for objective screening of cardiomegaly and longitudinal monitoring of cardiac size in captive bears, enhancing the clinical management and welfare of captive bears.

## Introduction

The Asiatic black bear (*Ursus thibetanus*, ABB) and sun bear (*Helarctos malayanus*, SB) are two of the eight extant bear species and are both listed as Vulnerable (VU) on the IUCN Red List [1,2]. Their populations are declining primarily due to poaching and illegal bile trade, as well as ongoing habitat loss and fragmentation [3–5]. Ongoing threats to wild populations, combined with the growing number of bears rescued from the illegal wildlife trade, have prompted the establishment of sanctuaries across several Asian countries. These facilities provide long-term care for individuals that cannot be released back into the wild [6]. In such sanctuaries, comprehensive annual health examinations are routinely conducted to maintain the bears’ well-being, with particular attention to age-related degenerative conditions, including cardiac, dental, and neoplastic diseases [7].

Among various chronic conditions observed in sanctuary populations, cardiovascular disease has been reported as a leading cause of mortality, particularly in bears formerly used for bile extraction [8]. These individuals often exhibit a high prevalence of systemic hypertension, with left ventricular hypertrophy being the most frequent cardiac lesion [9]. Consequently, routine monitoring for clinical signs of cardiac disease is essential for rescued bears. Among the manifestations of cardiac disease, changes in overall heart size are particularly relevant, as they can be readily evaluated through non-invasive diagnostic imaging. Cardiomegaly is a hallmark structural manifestation of cardiovascular disease; for instance, chronic systemic hypertension frequently results in pressure-overload–induced left ventricular hypertrophy [10]. Therefore, the assessment of cardiac silhouette size via thoracic radiography remains a fundamental and highly accessible diagnostic parameter in clinical settings [11].

The vertebral heart scale (VHS) was developed as an objective measure of cardiac size that is easily calculated from thoracic radiographs [12]. Its clinical utility lies in its ability to distinguish healthy individuals from those with cardiac disease and to monitor longitudinal cardiac changes within the same individual. While widely applied in domestic dogs and cats to evaluate cardiomegaly and associated comorbidities [13–17], VHS reference intervals (RI) are known to be species-specific. Consequently, RIs have been established for various wildlife species, including cheetahs, Humboldt penguins, fruit bats, raccoon dogs, Siberian weasels, aye-ayes, Rhesus macaques, and African wild dogs [18–25]. However, standardized reference data for assessing cardiac size or detecting enlargement in bears remain unavailable. This lack of reference values is particularly problematic in large mammals, where the clinical diagnosis of cardiomegaly is often constrained by logistical challenges, making the establishment of species-specific VHS ranges especially valuable for practical veterinary management.

The primary aim of this study was to establish radiographic VHS reference ranges for ABBs and SBs to characterize normal cardiac dimensions in both species. We first compared VHS values between species, then assessed thoracic morphology to provide anatomical context for interpreting radiographic measurements. Finally, we examined associations of biological factors—including sex, age, and morphometric measures (body weight, body condition score [BCS], and chest circumference)—with individual variation in VHS within each species.

## Materials and methods

### Animals and examination

This retrospective study included radiographs from 22 ABB and 24 SB housed at sanctuaries supported by the international animal welfare and wildlife conservation organization Free the Bears in Cambodia, Laos and Vietnam between 2022 and 2025. The organization works with local government partners to provide suitable housing for more than 300 bears rescued from the illegal wildlife trade.

Only bears deemed clinically healthy without cardiac or pulmonary diseases were included in the study. Health status was determined based on medical history, physical examination, complete blood count (CBC) and serum biochemistry analyses, and abdominal ultrasonography. All bears were anesthetized via remote intramuscular injection using a blow dart in Vietnam and Cambodia and a CO_2_-powered pistol in Laos, administering medetomidine (0.0125 mg/kg) and Zoletil® 100 (1.25 mg/kg). Following immobilization, bears were transported to the sanctuary clinic, where anesthesia was maintained with isoflurane via endotracheal intubation and supplemental oxygen at 2 L/min. All clinical procedures, including physical examination and radiographic examinations, were completed under general anesthesia within 1–2 hours.

As part of the physical examination, body weight and body condition score (BCS) were assessed at every health check before thoracic radiographs were taken. Body weight was measured using MP600 load bars (Datamars Ltd., Auckland, New Zealand) upon arrival at the clinic. BCS was evaluated using a bear-specific 5-point scale by veterinarians at each sanctuary who routinely conducted health examinations. Chest circumference was measured with the bears in ventral recumbency using a measuring tape placed immediately caudal to the forelimbs. During the health check, blood pressure was measured every 10 minutes. In addition, ophthalmic and funduscopic examinations were performed to assess retinal and other ocular vascular abnormalities associated with hypertension.

CBC was analyzed using site-specific hematology analyzers (Laos: XN-1000, Sysmex, Kobe, Japan; Cambodia: Pentra 80XL, HORIBA, Kyoto, Japan; Vietnam: ProCyte Dx, IDEXX Laboratories, Westbrook, ME, USA). Serum biochemistry was analyzed using the same point-of-care analyzer across all sites (VETSCAN® VS2, Zoetis, Parsippany, NJ, USA). Values for all measured parameters fell within reference intervals reported in previous studies of ABBs and SBs [26–28].

### Thoracic radiography

Thoracic radiographs were obtained at each site using locally available radiographic systems manufactured by different vendors (Laos: CR 15-X, Agfa-Gevaert NV, Mortsel, Belgium; Cambodia: ORANGE 1040HF, EcoRay Co., Ltd., Seoul, South Korea; Vietnam: TW-110, Samil X-ray, Koyang, South Korea). Radiographic exposure settings included a tube current of 4 mA for ABBs and 3.2 mA for SBs, with a tube voltage of 100 kVp for both species. ABB and SB were radiographed during inspiration in two standard projections: right lateral (RL) and dorsoventral (DV). DV views were selected over VD views because VD positioning results in greater image magnification due to the increased distance between the heart and the cassette [12]. Only radiographs without rotation, and with adequate visualization of thoracic structures were included in the analysis. VHS and thoracic morphology measurements were performed by a single observer using a commercially available DICOM viewer (Radiant DICOM Viewer, Medixant, Poznan, Poland), following the standard protocol originally described for canine [12].

### Radiographic measurements

**Fig 1:**
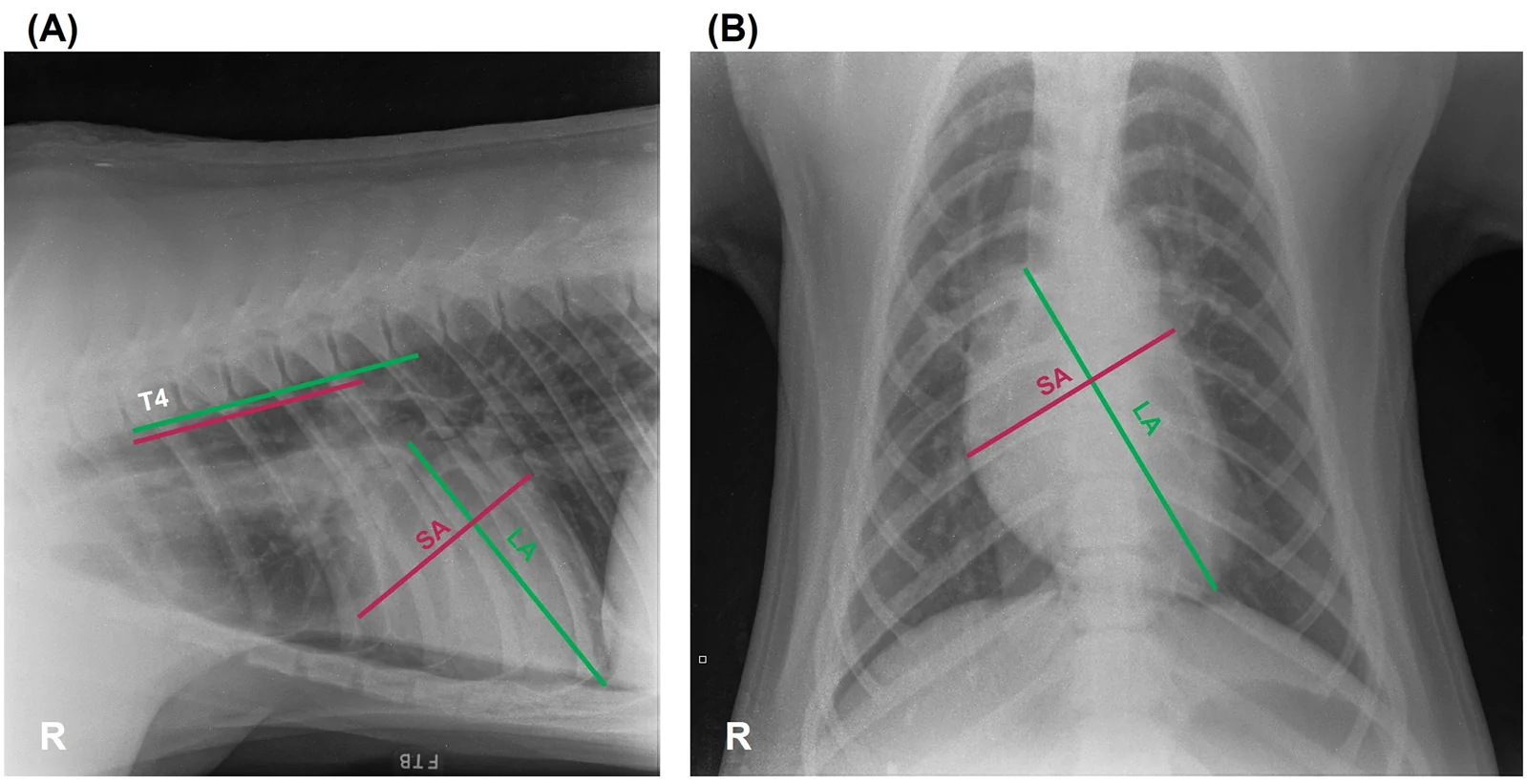
Measurements of the vertebral heart scale (VHS) in thoracic radiographs of an Asiatic black bear. (A) Right lateral measurement of the VHS using the long axis (LA) and short axis (SA) of the heart transposed onto the vertebral column beginning at the cranial edge of the fourth thoracic vertebra (T4). (B) Dorsoventral measurement of the VHS using the LA and SA of the heart.

VHS was calculated by measuring the cardiac long axis (LA) and short axis (SA) on thoracic radiographs and normalizing these values to vertebral length to minimize inter-individual variation in body size. Specifically, the measured LA and SA dimensions were transposed onto the vertebral column beginning at the cranial edge of the fourth thoracic vertebra (T4), and the total vertebral units were summed to yield the VHS value. On RL projections, the LA was measured from the carina of the bronchi to the cardiac apex, while the SA was measured perpendicular to the LA at the widest dimension of the cardiac silhouette (Fig 1A). On DV projections, the LA was defined as the maximal distance from the cardiac base to the apex, and the SA was measured as the longest line perpendicular to the LA (Fig 1B). Due to the large body size of bears, the T4 and the cardiac silhouette could not be fully visualized on a single radiograph; therefore, T4 length and cardiac dimensions were measured on separate images obtained during the same imaging session.

**Fig 2:**
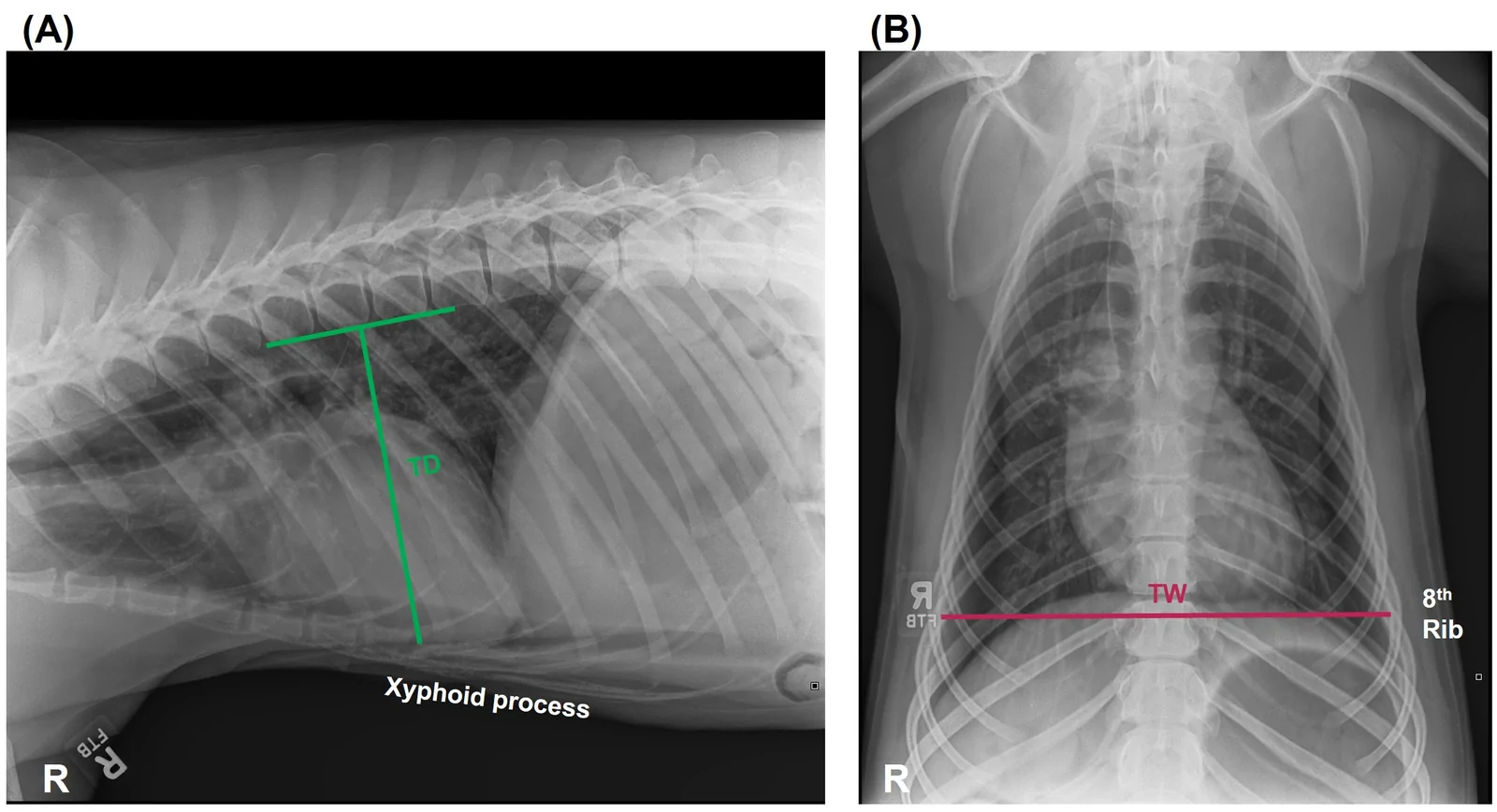
Measurements of the depth-to-width ratio (D/W ratio) in thoracic radiographs of a sun bear. (A) Measurement of the thoracic depth (TD) in right lateral radiography, defined as the distance from the cranial edge of the xiphoid process to the ventral border of the vertebral column. (B) Measurement of thoracic width (TW) in dorsoventral radiography as the maximal transverse distance between the medial borders of the eighth ribs.

Thoracic morphology was assessed using the ratio of thoracic depth to thoracic width, a metric commonly applied to characterize thoracic conformation. Thoracic depth was measured on RL projections as the distance from the cranial edge of the xiphoid process to the ventral border of the vertebral column, along a line drawn perpendicular to the vertebral axis (Fig 2A). Thoracic width was measured on DV projections as the maximal transverse distance between the medial borders of the eighth ribs (Fig 2B).

### Statistical analyses

Statistical analyses were performed using IBM SPSS Statistics for Windows (version 28.0; IBM Corp., Armonk, NY, USA). For all variables including species, sex, age, and body parameters, the mean, standard deviation (SD), and coefficient of variation (CV) were calculated. Reference ranges were nonparametrically defined as the 2.5th–97.5th percentiles. Normality was assessed using the Shapiro–Wilk test and visually inspected using Q–Q plots. As all variables satisfied the assumption of normality, homogeneity of variances was evaluated using Levene’s test. Comparisons of VHS and thoracic depth-to-width (D/W) ratio between species were performed. Within each species, differences according to sex and age group were evaluated using two-sample t-tests. Associations with morphometric parameters were assessed using multiple linear regression analysis. Depending on the results of the variance homogeneity test, Student’s t-test was used for equal variances and Welch’s t-test for unequal variances. Multiple linear regression analyses were used to evaluate the relationships between RL VHS and morphometric parameters, including body weight, BCS, and chest circumference, while adjusting for sex. Analyses were performed using R version 4.5.2 (accessed on 26^th^ January 2026).

## Results

A total of 25 ABBs and 30 SBs were initially identified during the study period. Of these, 22 ABBs and 24 SBs met the criteria for the final radiographic analysis. Sample sizes varied across analyses due to the exclusion of radiographs with inadequate positioning or individuals with unknown age. The ABB group consisted of 11 males and 11 females, including 6 juveniles (<5 years), 13 adults (5–21 years), and 3 individuals of unknown age. The SB group consisted of 8 males and 16 females, including 3 juveniles, 20 adults (6–28 years), and 1 individual of unknown age. The sampled bears were rescued average age at 1.1 years, and had been in sanctuary care for an average of approximately 9.9 years prior to radiography (range, 0.2–20.7 years). Reproductive status varied among individuals because of management practices and was not controlled for in the present study. Morphometric characteristics of the study population are provided in Table 1.

**Table 1:** Morphometric characteristics of Asiatic black bears and sun bears included in the study.

Variable	Asiatic black bear			Sun bear		
	n	Mean ± SD	Range	n	Mean ± SD	Range
Weight (kg)	22	125.86 ± 35.90	73.00 ‒ 193.00	24	60.65 ± 8.67	42.00 ‒ 73.00
BCS	22	3.14 ± 0.62	2.00 ‒ 5.00	24	2.85 ± 0.45	2.00 ‒ 4.00
Chest circumference (cm)	22	108.07 ± 13.99	86.00 ‒ 139.30	24	86.07 ± 8.79	72.00 ‒ 113.60

Abbreviations: n, number of subjects; SD, standard deviation; BCS, body condition score (5-point scale).

In ABBs, the mean RL VHS was 10.65 ± 0.48 vertebrae (v) and the mean DV VHS was 10.82 ± 0.73 v (Table 2), whereas in SBs, the mean RL VHS was 10.68 ± 0.62 v and the mean DV VHS was 11.18 ± 0.67 v (Table 3). No differences in RL or DV VHS values were detected between ABBs and SBs using Student’s t-test.

**Table 2:** Radiographic cardiac measurements and reference ranges for Asiatic black bears (*Ursus thibetanus*).

Variable	n	Mean ± SD	Median	95% Reference range
RL VHS (v)	20	10.65 ± 0.48	10.66	9.90 ‒ 11.67
RL T4 (mm)	20	30.72 ± 3.71	30.20	25.00 ‒ 35.80
RL LA (mm)	20	183.69 ± 23.85	183.35	146.90 ‒ 215.40
RL SA (mm)	20	143.65 ± 20.24	142.10	116.60 ‒ 175.60
DV VHS (v)	17	10.82 ± 0.73	10.92	8.90 ‒ 11.96
DV T4 (mm)	17	31.08 ± 3.63	31.40	24.80 ‒ 35.70
DV LA (mm)	17	195.93 ± 30.94	188.30	128.80 ‒ 239.20
DV SA (mm)	17	139.88 ± 21.89	138.80	108.00 ‒ 190.70

Abbreviations: n, number of subjects; SD, standard deviation; RL, right lateral; VHS, vertebral heart scale; v, vertebrae; T4, fourth thoracic vertebra; LA, cardiac long axis; SA, cardiac short axis; DV, dorsoventral.

**Table 3:** Radiographic cardiac measurements and reference ranges for sun bears (*Helarctos malayanus*).

Variable	n	Mean ± SD	Median	95% Reference range
RL VHS (v)	22	10.68 ± 0.62	10.71	9.19 ‒ 11.68
RL T4 (mm)	22	24.80 ± 2.62	24.95	20.00 ‒ 30.50
RL LA (mm)	22	147.92 ± 15.00	148.65	118.90 ‒ 178.90
RL SA (mm)	22	116.25 ± 11.24	116.30	92.50 ‒ 134.70
DV VHS (v)	20	11.18 ± 0.67	11.15	10.13 ‒ 12.28
DV T4 (mm)	20	25.39 ± 2.34	25.45	20.70 ‒ 30.50
DV LA (mm)	20	168.15 ± 15.23	167.80	143.40 ‒ 195.20
DV SA (mm)	20	114.91 ± 9.15	113.40	98.80 ‒ 136.30

Abbreviations: n, number of subjects; SD, standard deviation; RL, right lateral; VHS, vertebral heart scale; v, vertebrae; T4, fourth thoracic vertebra; LA, cardiac long axis; SA, cardiac short axis; DV, dorsoventral.

Thoracic morphology showed distinct values between ABBs and SBs, as reflected by measurements of thoracic depth, thoracic width, and the D/W ratio (Table 4). In ABBs, the mean D/W ratio was 0.84 ± 0.08, whereas in SBs, the mean D/W ratio was 0.69 ± 0.05. Significant interspecific differences were detected in D/W ratio and thoracic depth values using Welch’s t-test (*P* < 0.05).

**Table 4:** Comparison of thoracic morphology parameters between Asiatic black bears (*Ursus thibetanus*) and sun bears (*Helarctos malayanus*).

Variable	Asiatic black bear			Sun bear		
	n	Mean ± SD	95% Reference range	n	Mean ± SD	95% Reference range
D/W ratio	14	0.84 ± 0.08^*^	0.73 ‒ 1.00	16	0.69 ± 0.05^*^	0.59 ‒ 0.77
TD (cm)	14	23.45 ± 4.01^*^	18.24 ‒ 31.69	16	17.55 ± 1.70^*^	14.95 ‒ 20.77
TW (cm)	14	28.05 ± 4.95	19.70 ‒ 34.86	16	25.49 ± 1.55	22.65 ‒ 28.28

Abbreviations: n, number of subjects; SD, standard deviation; D/W ratio, depth-to-width ratio; TD, thoracic depth; TW, thoracic width.

* *P* < 0.05

VHS and D/W ratio values were compared according to sex and age in ABBs and SBs (Tables 5 and 6). In both species, no differences in RL or DV VHS values were detected between males and females or between adults and juveniles. Similarly, D/W ratio did not differ according to sex or age in either species.

**Table 5:** Comparison of vertebral heart scale values and depth-to-width ratio by sex and age in Asiatic black bears (*Ursus thibetanus*).

Variable	Sex			Age		
	n (M, F)	Male	Female	n (A, J)	Adult	Juvenile
RL VHS (v)	9, 11	10.86 ± 0.38	10.49 ± 0.47	11, 6	10.70 ± 0.46	10.38 ± 0.35
DV VHS (v)	10, 7	11.10 ± 0.54	10.42 ± 0.85	13, 4	10.79 ± 0.83	10.79 ± 0.70
D/W ratio	8, 6	0.86 ± 0.07	0.83 ± 0.09	10, 4	0.83 ± 0.08	0.86 ± 0.07

Values are presented as mean ± SD.

Abbreviations: n, number of subjects; M, male; F, female; A, adult; J, juvenile; RL, right lateral; VHS, vertebral heart scale; v, vertebrae; DV, dorsoventral; D/W ratio, depth-to-width ratio.

**Table 6:** Comparison of vertebral heart scale values and depth-to-width ratio by sex and age in sun bears (*Helarctos malayanus*).

Variable	Sex			Age		
	n (M, F)	Male	Female	n (A, J)	Adult	Juvenile
RL VHS (v)	7, 15	10.64 ± 0.77	10.70 ± 0.57	19, 3	10.64 ± 0.62	10.61 ± 0.47
DV VHS (v)	7, 13	11.27 ± 0.60	11.13 ± 0.72	17, 3	11.19 ± 0.70	11.16 ± 0.58
D/W ratio	6, 10	0.72 ± 0.04	0.67 ± 0.05	13, 3	0.64 ± 0.05	0.70 ± 0.05

Values are presented as mean ± SD.

Abbreviations: n, number of subjects; M, male; F, female; A, adult; J, juvenile; RL, right lateral; VHS, vertebral heart scale; v, vertebrae; DV, dorsoventral; D/W ratio, depth-to-width ratio.

**Fig 3:**
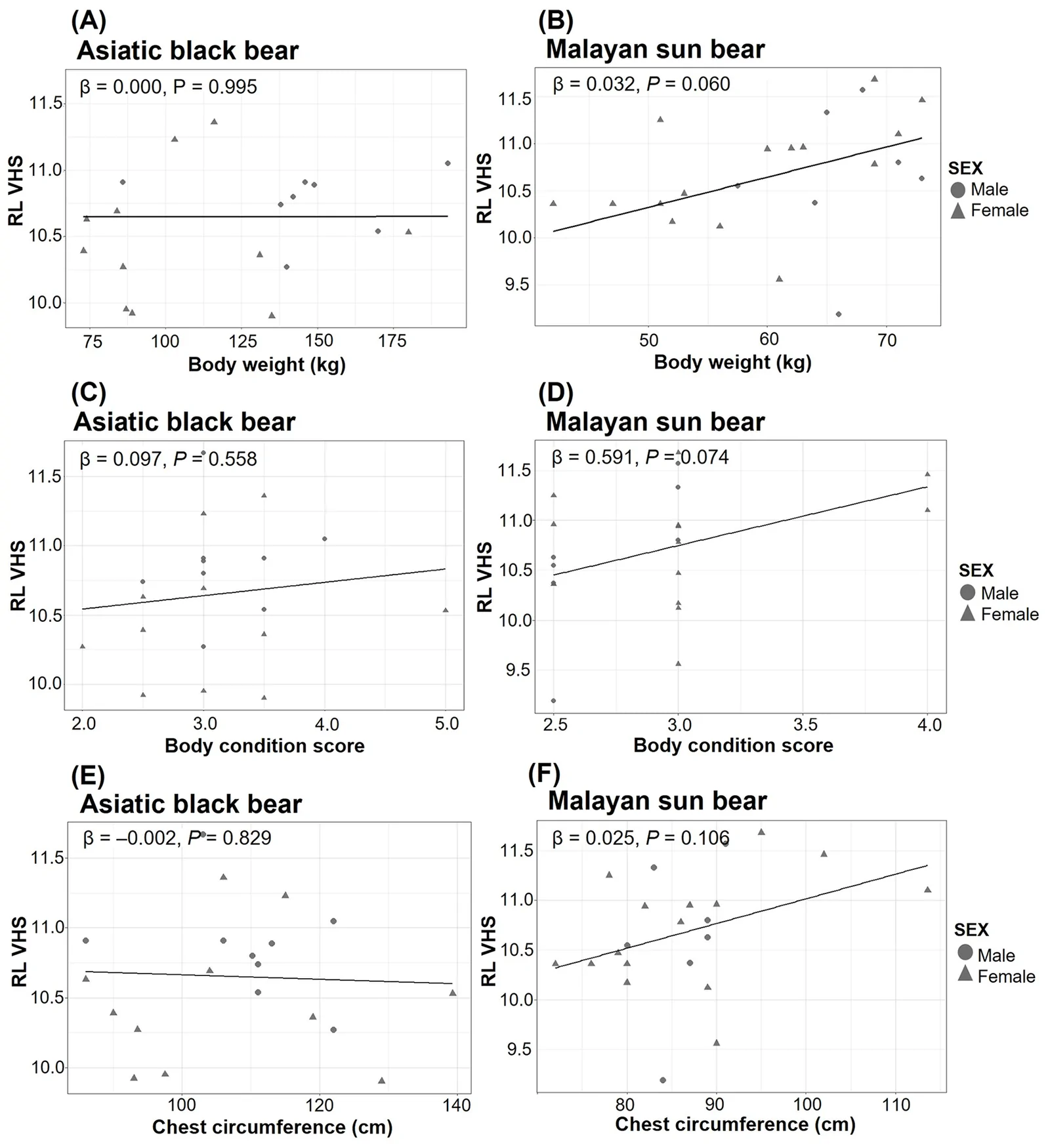
Sex-adjusted linear regression between morphometric parameters and right lateral vertebral heart scale in Asiatic black bears (ABBs, n = 20) and sun bears (SBs, n = 22). This analysis examines whether morphometric parameters are associated with right lateral vertebral heart scale, independent of differences between males and females. Black lines indicate sex-adjusted mean fitted values from a multiple linear regression model. (A, B) Body weight, (C, D) body condition score, and (E, F) chest circumference in ABBs and SBs, respectively.

In the multiple linear regression analyses adjusted for sex, body weight, BCS, and chest circumference showed no association with RL VHS in either species. However, SBs showed non-significant increases in RL VHS across all body parameters (Fig 3). Specifically, body weight had no effect on RL VHS in ABBs (β = 0.000; *P* = 0.995; adjusted *R*^*2*^ = 0.076), while SBs exhibited a non-significant positive coefficient (β = 0.032; *P* = 0.060; adjusted *R*^*2*^ = 0.090). BCS showed positive but non-significant coefficients in both species (ABBs: β = 0.097, *P* = 0.558, adjusted *R*^*2*^ = 0.095; SBs: β = 0.591, *P* = 0.074, adjusted *R*^*2*^ = 0.073). Chest circumference showed a non-significant negative coefficient in ABBs (β = –0.002; *P* = 0.829; adjusted *R*^*2*^ = 0.079) and a non-significant positive coefficient in SBs (β = 0.025; *P* = 0.106; adjusted *R*^*2*^ = 0.043).

## Discussion

This study represents the first VHS reference ranges in bear species and to comparison of VHS values across species, sex, age, and morphometric parameters. In our findings, the mean RL VHS values for ABBs and SBs were 10.65 ± 0.48 v and 10.68 ± 0.62 v, respectively, while the DV VHS values were 10.82 ± 0.73 v and 11.18 ± 0.67 v. RL VHS values exhibited a lower SD than DV VHS values in both species, likely reflecting a higher risk of cardiac silhouette distortion in the DV view. Accordingly, the RL view may offer a more reliable and reproducible approach for VHS measurement by minimizing potential measurement errors.

Previous studies have demonstrated that VHS reference ranges are highly species-specific, as evidenced by the reported RL values in diverse taxa: 9.7 ± 0.5 v in dogs [12], 8.2 ± 0.9 v in cheetahs [18], 6.7 ± 0.6 v in Siberian weasels [22], and 9.13 ± 0.42 v in buffaloes [29]. These reference ranges appear to be independent of absolute body size or weight, reflecting instead the unique anatomical and physiological adaptations of each species. These interspecific differences underscore the necessity of establishing baseline reference values for each species individually to ensure clinical precision in assessing cardiac health.

Although the mean RL and DV VHS values were slightly higher in SBs than in ABBs, the differences were not statistically significant. Previous studies have reported variation in VHS among closely related taxa, such as between different dog breeds [14,15] and between congeneric mustelid species, specifically the Siberian weasel and the ferret [22,30]. In contrast, our findings suggest that VHS values remain comparable between ABBs and SBs, despite their classification into different genera. Further investigation is warranted to determine whether this similarity in relative cardiac scale is consistent across other members of the family Ursidae.

In contrast to the consistent VHS values, the thoracic morphology—assessed by the D/W ratio—showed significant differences between ABBs (0.84 ± 0.08 v) and SBs (0.69 ± 0.05 v). Specifically, the D/W ratios reported for ABBs fall within the range commonly described in other mammals for which reference data are available, such as dogs, raccoon dogs, and aye-ayes (0.75–1.25) [14,21,23], whereas SBs showed markedly lower values (0.64–0.74). This suggests that while the relative cardiac size compared to vertebral length is conserved, the thoracic conformation differs markedly: ABBs possess a deeper (RL view) and narrower (DV view) thorax, whereas SBs have a shallower and broader one. Such morphological variations may reflect evolutionary adaptations to their respective ecological niches. Although both species are capable climbers and utilize arboreal resources, the comparatively broader thorax in SBs may be associated with climbing-relate adaptations, together with forelimb conformations such as slight inward bowing [31]. Consequently, our findings indicate that despite these evolutionary differences in thoracic shape, the VHS remains a consistent and reliable metric for comparing relative cardiac size between these two species.

Sex, age, body weight, BCS, and chest circumference were not associated with VHS in either bear species. This pattern aligns with previous findings in dogs [12], cats [16], and other wildlife [21–23], supporting VHS as a relatively stable radiographic parameter across individuals. In regression analyses, none of the morphometric variables were significantly associated with VHS; however, positive but non-significant coefficients were observed in SBs. Unlike a prior canine study suggesting a potential effect of BCS on VHS [15], BCS was not associated with VHS in either ABBs or SBs in the present study. Overall, the regression models explained little variance (adjusted *R²* < 0.10 for all variables), indicating that morphometric traits contribute minimally to inter-individual variation in VHS. Clinically, this is advantageous for sanctuary populations in which body condition and thoracic conformation can vary widely due to diverse backgrounds (e.g., pet trade, wild rescue, or long-term captivity), allowing cardiac silhouette size to be interpreted without correction factors and supporting both cross-sectional evaluation and longitudinal monitoring in field settings.

The marked difference in body weight ranges between the two species—73–193 kg in ABBs and 42–73 kg in SBs—provides additional insight into the observed pattern. In ABBs, the absence of any detectable association between VHS and body weight, despite a wide range of body mass, indicates that VHS is a robust metric capable of normalizing cardiac size across substantial inter-individual variation. In contrast, RL VHS tended to increase with body weight in SBs, although this was not statistically significant (*P* = 0.060). This pattern, observed within a relatively narrow body size range, suggests that VHS may be more sensitive to physiological variations in smaller ursids. While this study focused on *H. malayanus malayanus*, further investigation in larger populations or comparative analyses incorporating the smaller subspecies, *H. m. euryspilus*, would be beneficial to clarify the potential influence of body size and subspecies-specific traits on radiographic cardiac parameters.

Several limitations of this study should be acknowledged. First, as thoracic radiographs were obtained across multiple regions by different personnel, some degree of inter-observer variability in radiographic positioning and technique may have occurred, despite the presence of standardized imaging protocols at the sanctuaries. Second, echocardiography—the gold standard for assessing cardiac structure and function [32]—could not be performed due to logistical constraints and equipment limitations. Consequently, the potential inclusion of bears with occult cardiac disease cannot be completely ruled out, as radiography has relatively low sensitivity for detecting such conditions [33]. Third, although blood pressure was measured during the health examinations, the cardiovascular effects of sedation may have influenced these values, precluding the definitive exclusion of concurrent hypertensive disease. Despite these limitations, all included individuals were clinically asymptomatic, and thoracic radiographs were carefully reviewed to exclude any signs of overt cardiac enlargement, pulmonary edema, or pleural effusion, thereby minimizing the potential inclusion of subjects with significant cardiac disease. Lastly, sample sizes for certain sex and age categories were relatively small, which may have limited the statistical power to detect subtle differences among these groups. Future studies involving larger and more balanced cohorts across age and sex categories are warranted to further refine species-specific reference ranges.

## Conclusion

This study established the first radiographic cardiac reference ranges for ABBs and SBs, showing that VHS reference ranges were comparable between species despite marked differences in thoracic conformation. The absence of significant association between VHS and biological traits (e.g., sex, age, and body weight) further supports VHS as a robust radiographic metric with minimal individual variability in both species. Accordingly, VHS appears to reflect intrinsic cardiac dimensions rather than general body conformation, enhancing its clinical utility in heterogeneous sanctuary populations. The species-specific VHS reference ranges established here may therefore provide a practical tool for detecting cardiomegaly and monitoring longitudinal changes in cardiac size at the individual level. While VHS is useful for screening, definitive diagnosis of cardiovascular disease still requires complementary assessments such as echocardiography and/or cardiac biomarkers. Overall, these findings offer a standardized baseline to support cardiac monitoring and clinical management of ABBs and SBs across diverse captive settings. This study thereby contributes to the long-term clinical management and improved welfare of these threatened species across diverse captive environments.

## Supporting information

S1 Data Rawdata. (XLSX)
